# The gastric carcinosarcoma with severe venous invasion: a case report

**DOI:** 10.1186/s40792-018-0421-8

**Published:** 2018-01-30

**Authors:** Tomoaki Bekki, Nobuaki Fujikuni, Kazuaki Tanabe, Shuji Yonehara, Hironobu Amano, Toshio Noriyuki, Masahiro Nakahara

**Affiliations:** 1 Department of Surgery, Onomichi General Hospital, Onomichi, Hiroshima Japan; 2 Department of Gastroenterological and Transplant Surgery, Applied Life Sciences, Institute of Biomedical and Health Sciences, Hiroshima University, Hiroshima, Japan; 3 Department of Pathology, Onomichi General Hospital, Onomichi, Hiroshima Japan

**Keywords:** Gastric carcinosarcoma, Severe venous invasion, Histogenesis, Immunohistochemical analysis

## Abstract

**Background:**

Gastric carcinosarcoma with severe venous invasion is extremely rare, and to the best of our knowledge, this is the first reported case.

**Case presentation:**

A 79-year-old man visited the Onomichi General Hospital following abnormal upper gastrointestinal series findings. Laboratory data demonstrated no anemia, and the serum carcinoembryonic antigen (CEA) and carbohydrate antigen 19-9 (CA19-9) levels were not elevated. Endoscopy identified a Borrmann type III lesion in the cardiac end of the stomach. Abdominal contrast-enhanced computerized tomography (CT) indicated that the lesser curvature of the stomach wall was modestly enhanced with bulky lymph nodes. Pathological biopsy examination identified a group 5, papillary adenocarcinoma. We diagnosed advanced gastric cancer with bulky lymph nodes (cT4aN3M0, cStage IIIC). Following neoadjuvant chemotherapy, the patient underwent open total gastrectomy with D2 lymph node dissection. Histopathologically, the tumor consisted of two components: a tubular adenocarcinoma and a sarcoma. The tumor cells were mainly intravenous and were not detected in the gastric wall stroma; this resulted in a venous invasion. Immunohistochemical analysis revealed that the tumor was positive for vimentin and partly positive for desmin and cytokeratin CAM5.2. We diagnosed a true gastric carcinosarcoma with severe venous invasion. Abdominal CT 2 months after surgery showed a low density area in the liver, suggesting metastasis.

**Conclusions:**

Carcinosarcomas with lymph node metastasis are sometimes reported, but progression into the vasculature is very rare. We present a case of carcinosarcoma with unusual progression characteristics.

## Background

Carcinosarcomas are biphasic tumors that are composed of both carcinomatous and sarcomatous cells [1], which are most frequently observed in the esophagus [2]; however, they have also been detected in other organs such as the uterus, breast, thyroid, lung, and upper gastrointestinal system [3]. Middle-aged and elderly men with a history of smoking or drinking are most likely to present with this disease [4]. Gastric carcinosarcoma shows no specific clinical symptoms and is similar to gastric carcinoma with respect to loss of appetite, dysphagia, abdominal pain, and anemia due to blood loss from the tumor [5].

Upper gastrointestinal series, abdominal contrast-enhanced computerized tomography (CT), and endoscopy demonstrate no specific findings and as such do not play an important role in determining the precise diagnosis [4]. The gold standard for the diagnosis of carcinosarcoma is immunohistochemistry. Effective therapy has not been established, and the only curative treatment is surgery for gastric carcinosarcoma without distant metastasis [6].

Gastric carcinosarcoma is rare [7] and has not been frequently reported. In addition, concurrent severe venous invasion is extremely rare, and to the best of our knowledge, this is the first reported case.

We present herein a surgical case of gastric carcinosarcoma with severe venous invasion.

## Case presentation

**Fig. 1:**
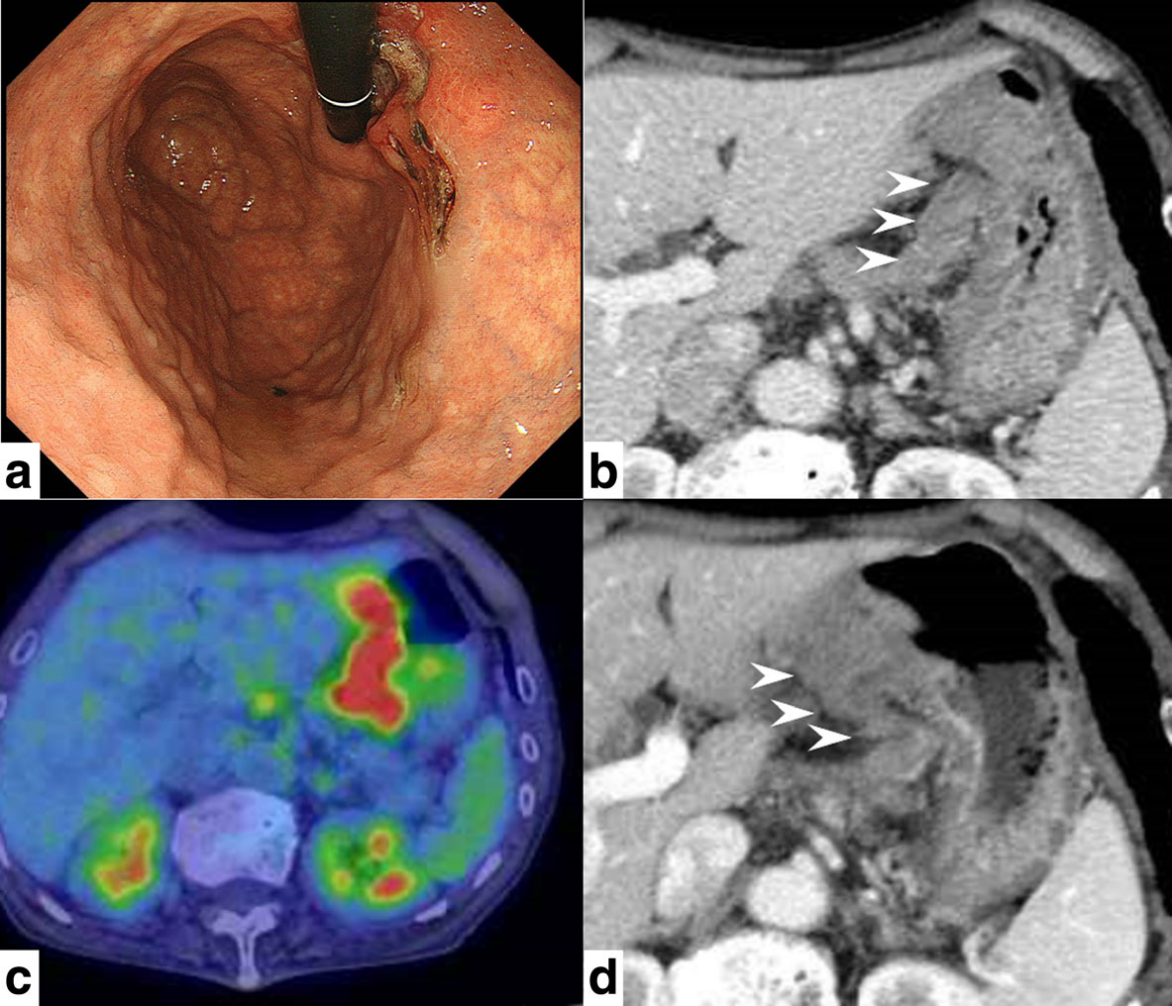
**a** Endoscopic findings. A Borrmann type III tumor was found in the cardiac end of the stomach and irregular wall thickening was identified in the lesser curvature. **b** Abdominal contrast-enhanced computed tomography (CT) findings. Abdominal contrast-enhanced CT indicated that the lesser curvature stomach wall was modestly enhanced with suspicious bulky lymph nodes in the lesser curvature (white arrowhead). **c** Positron emission tomography (PET) findings. PET revealed an accumulation of fludeoxyglucose (18F) in the stomach wall and bulky lymph nodes were detected by computed tomography (CT). **d** Abdominal contrast-enhanced computed tomography (CT) findings after preoperative chemotherapy. Abdominal contrast-enhanced CT demonstrated that the size of tumor and bulky lymph nodes had not changed (white arrowhead)

**Fig. 2:**
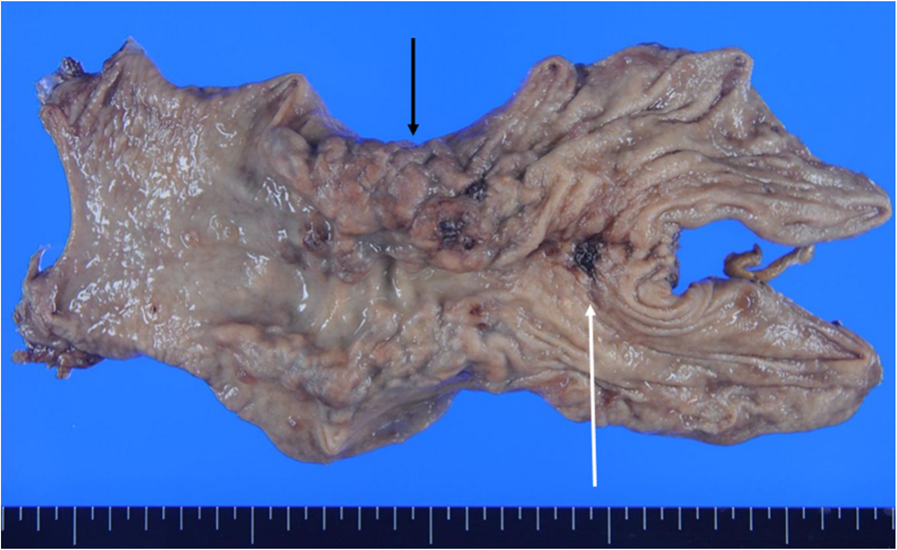
Macroscopic findings. A specimen of tumor 15 × 8 cm in size, analogous to the polypoid tumor (Borrmann type III) expanded over the lesser curvature (black arrow), and an ulcer in the immediate cardiac end of the lesser curvature posterior wall (white arrow)

**Fig. 3:**
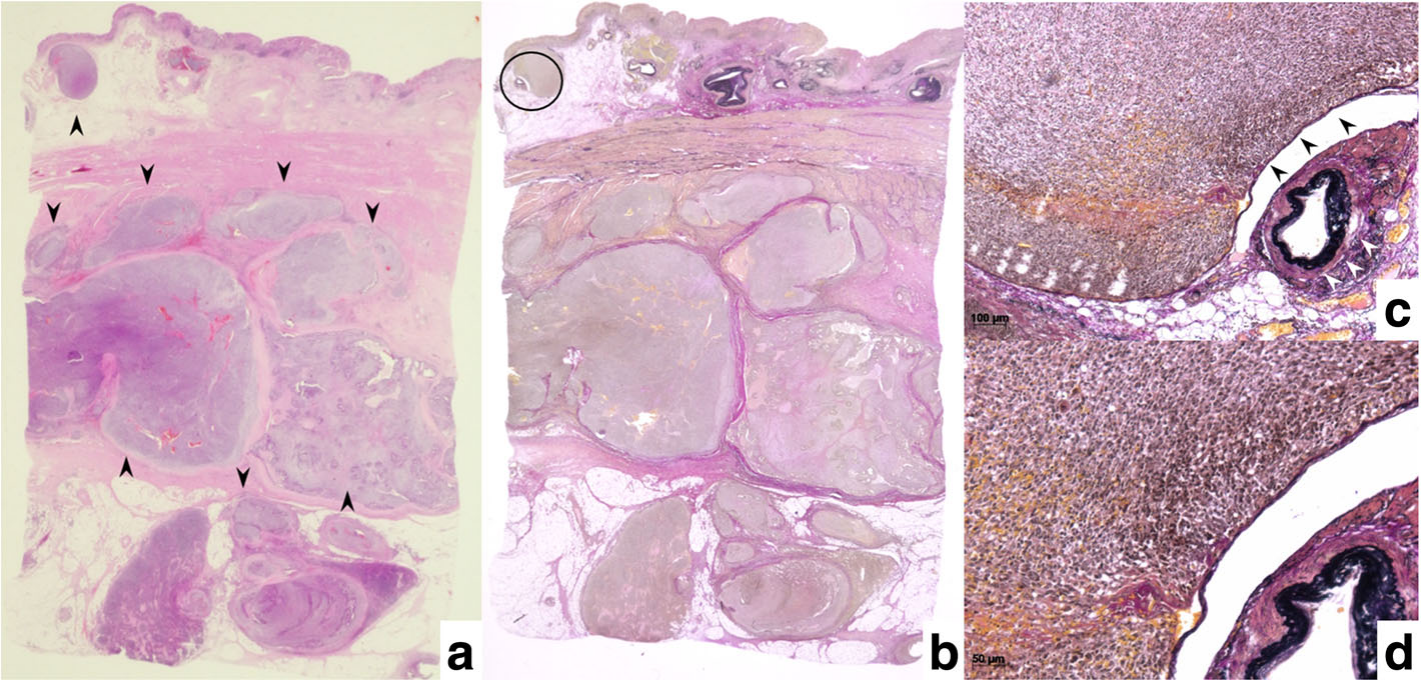
Macroscopic pathology findings. **a**, **b** The tumor cells were mainly found intravenously inside and outside of the stomach wall (black arrowhead) and were not found in the gastric wall stroma. **a** Hematoxylin-eosin stain, original magnification. **b** Elastic van Gieson staining, original magnification. **c**, **d** These are pictures enlarged with a circle on **b**. The tumor cells existed in venous (black arrowhead) locations and could be identified among the elastic fibers of veins and nearby arteries (white arrowhead). **c** Elastic van Gieson staining, original magnification × 50. **d** Elastic van Gieson staining, original magnification × 100

**Fig. 4:**
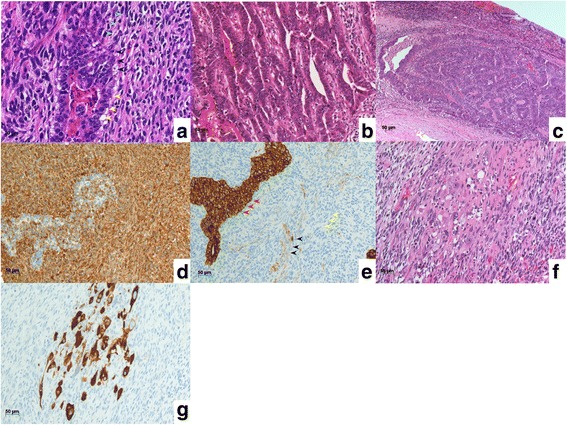
Histopathological findings. **a** Transitional zones (black arrowhead) between the carcinomatous (yellow arrowhead) and sarcomatous components (green arrowhead) were observed in the tumor. **b** The adenocarcinoma component was found in the mucosa of the stomach. **c** The venous invasion found in the submucosa consisted of adenocarcinoma cells. Immunohistochemical findings. **d** The tumor was positive for vimentin. **e** The carcinomatous components (red arrowhead) were positive for cytokeratin CAM5.2 and the sarcomatous components (yellow arrowhead) were partly positive for cytokeratin CAM5.2 (black arrowhead). Histopathological findings of rhabdomyosarcoma. **f** The tumor contained cells with eosinophil-abundant cytoplasm. **g** The cells with eosinophil-abundant cytoplasm were positive for desmin

A 79-year-old man was admitted to the Department of Surgery at the Onomichi General Hospital following abnormal upper gastrointestinal series findings. He did not have any complaints such as epigastric pain, nausea, or weight loss. He had no comorbidities and his previous surgical history included right cataract removal. Laboratory data showed that his white blood cell (WBC) count and C-reactive protein (CRP) levels were within the normal range. He had no anemia and his serum carcinoembryonic antigen (CEA) and carbohydrate antigen 19-9 (CA19-9) levels were not elevated. Endoscopic findings showed a Borrmann type III lesion in the cardiac end of the stomach and irregular wall thickening in the lesser curvature (Fig. 1a). Abdominal contrast-enhanced CT indicated that the lesser curvature was modestly enhanced, and bulky lymph nodes were detected (Fig. 1b). Positron emission tomography (PET) revealed an accumulation of fludeoxyglucose (18F) in the stomach wall, and bulky lymph nodes were detected by CT (Fig. 1c). The pathological findings led to a diagnosis of group 5, papillary adenocarcinoma. He was diagnosed with advanced gastric cancer with bulky lymph nodes (cT4aN3M0 cStage IIIC) and underwent 4 cycles of preoperative chemotherapy using tegafur/gimeracil/oteracil (S-1) and oxaliplatin. Abdominal contrast-enhanced CT after preoperative chemotherapy demonstrated that the size of the tumor and bulky lymph nodes remained unchanged (Fig. 1d). Endoscopic examination revealed that the tumor had expanded and laparoscopic examination did not identify the presence of peritoneal dissemination. The patient subsequently underwent open total gastrectomy with D2 lymph node dissection. The total surgery time was 258 min and the total intraoperative bleeding volume was 350 ml. Macroscopically, a specimen of tumor measuring 15 × 8 cm was analogous to the polypoid tumor (Borrmann type III) that was found in the lesser curvature. An ulcer was also identified in the cardiac end of the posterior wall in the lesser curvature (Fig. 2). Histopathologically, the tumor was diagnosed as carcinosarcoma consisting of both carcinomatous and sarcomatous components. Lymph node metastasis was not identified. The lymph nodes had no tumor cells nor necrotic changes of tumor cells, but severe venous invasion was detected. The tumor cells were mainly intravenous inside and outside of the stomach wall; however, they were not detected in the stomach wall stroma (Fig. 3a–d). There were mainly spindle cells identified with some adenocarcinoma cells. The tumor had transitional zones between the carcinomatous and sarcomatous components (Fig. 4a) and contained cells with eosinophil-abundant cytoplasm. The adenocarcinoma component was detected in the mucosa of the stomach where we also identified a venous invasion. The invasion consisted of adenocarcinoma cells from the submucosa (Fig. 4b, c). Immunohistochemical staining revealed that the tumor was positive for vimentin and partly positive for cytokeratin CAM5.2 (Fig. 4d, e). The tumor had a rhabdomyosarcoma component, identified by the presence of cells with eosinophil-abundant cytoplasm that were positive for desmin (Fig. 4f, g). The patient was diagnosed with true gastric carcinosarcoma with severe venous invasion; both the proximal and distal margins were negative.

**Fig. 5:**
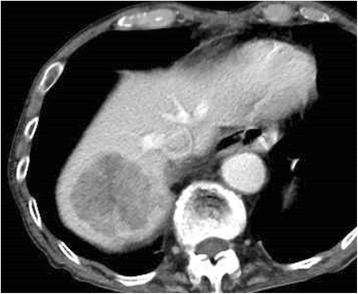
Abdominal computed tomography (CT) findings 2 months after surgery. A liver tumor was identified with enhanced delivery at the edges implying high external density and low intracellular density

Abdominal CT 2 months after surgery showed a low-density area in the liver, which was a suspected metastasis. A month later, the low-density area increased. Abdominal contrast-enhanced CT revealed a low intracellular density and ring enhancement (Fig. 5).

## Discussion

Carcinosarcomas are classified into two subtypes: true carcinosarcoma and so-called carcinosarcoma. It has been reported that true carcinosarcomas have three histological features [8]. The first is the presence of genuine sarcomatous components, such as chondrosarcoma, osteosarcoma, rhabdomyosarcoma, and leiomyosarcoma. Second, the tumors contain no traditional zones between the carcinomatous and sarcomatous components. Finally, the sarcomatous component is positive for mesenchymal markers and negative for epithelial markers. Immunohistochemical analysis is the gold standard for the diagnosis of carcinosarcoma. CEA, epithelial membrane antigen (EMA), pancreatin, chromogranin A, CD56, and synaptophysin are specific markers to identify carcinomatous components, whereas desmin, vimentin, and α-smooth muscle/sarcometic actin are specific makers to identify sarcomatous components [9]. Ro et al. proposed additional histological criteria for the diagnosis of carcinosarcomas [10]. First, epithelial tumor cells and spindle cells exist concurrently, and there is a transitional area between the two components. The second criterion is that the sarcomatoid component exhibits an epithelial phenotype. Consequently, the definition of true carcinosarcoma is unclear and it remains controversial whether the lesion must satisfy all of the above histological criteria [8]. In our case, the tumor contained transitional zones between the adenocarcinoma and sarcomatous components in some places and had biphasic components of both adenocarcinoma and sarcoma. The carcinomatous components were positive for cytokeratin CAM5.2, and the sarcomatous components were positive for vimentin and partly positive for cytokeratin CAM5.2. Additionally, the tumor contained a genuine sarcomatous component, rhabdomyosarcoma. Therefore, the patient was diagnosed with true gastric carcinosarcoma.

In the upper gastrointestinal tract, carcinosarcomas are frequently observed in the esophagus [2]. However, gastric carcinosarcoma is extremely rare [7] and has been less frequently reported. Therefore, the histogenesis and progression of this tumor remain unclear. Two hypotheses have been proposed [11]. The first is the biclonal origin hypothesis, which states that carcinosarcomas originate from two different tumor cell clones. The second is the monoclonal origin hypothesis, which states that the carcinosarcoma may originate from a stem cell, which is capable of undergoing both epithelial and mesenchymal differentiation. The tumor in our case probably originated as an epithelial tumor because there was an adenocarcinoma identified in the mucosa of the stomach. However, the tumor cells were mainly intravenous inside and outside of the stomach wall and the main component was spindle cells. Immunohistochemistry revealed that the sarcomatous components were partly positive for cytokeratin CAM5.2. It is known that sarcoma can be occasionally positive for epithelial markers; therefore, positive staining for some epithelial markers does not prove that sarcomatous components originated as carcinomatous components. In our case, the sarcomatous components were partly positive for epithelial makers and there was a transitional area between the carcinomatous and sarcomatous components. A similar case was reported in the past [7]; therefore, we suggest that the tumor originated in the mucosa of the stomach as an epithelial tumor and later transformed into a carcinosarcoma. As such, our findings support the hypothesis that carcinosarcomas may originate from a stem cell.

In this case, the tumor cells were mainly intravenous inside and outside of the stomach wall; however, tumor cells were not found in the stomach wall stroma. In addition, the adenocarcinoma component formed a severe venous canal invasion in the submucosa showing that the tumor had progressed into the vasculature. It can be inferred that the reason for early postoperative liver metastasis was severe venous invasion. The bulky lymph nodes in the lesser curvature identified by preoperative abdominal CT also suggest that the tumor progressed into the vasculature. The bulky mass along the lesser curvature of the stomach identified by preoperative CT turned out to be the vein involved with tumor cells. The biopsy suggested papillary adenocarcinoma; this may be because the tumor cells in the mucosa showed carcinomatous components, and we took mucosa from the stomach where only carcinomatous components could be found. As such, this case was very difficult case to diagnose preoperatively.

Gastric carcinosarcomas have very poor prognosis. Gastric carcinosarcomas have a low incidence of metastasis; however several cases have occurred wherein liver metastasis was detected [9, 12]. There is uncertainty surrounding the pathogenesis of gastric carcinosarcoma, and this represents the first case of gastric carcinosarcoma with severe venous invasion, to our knowledge.

## Conclusions

We identified a rare case of gastric carcinosarcoma that showed a specific progression pattern, which necessitates further study of gastric carcinosarcoma.

## Abbreviations

CA19-9: Carbohydrate antigen 19–9
CEA: Carcinoembryonic antigen
CRP: C-reactive protein
CT: Computed tomography
EMA: Epithelial membrane antigen
PET: Positron emission tomography
S-1: Tegafur/gimeracil/oteracil
WBC: White blood cell

## References

[1] Wick MR, Swanson PE (1993). Carcinosarcomas: current perspectives and an historical review of nosological concepts. Semin Diagn Pathol.

[2] Chino O, Kijima H, Shimada H (2000). Clinicopathological studies of esophageal carcinoma in achalasia: analyses of carcinogenesis using histological and immunohistochemical procedures. Anticancer Res.

[3] Robey-Cafferty SS, Grignon DJ, Ro JY (1990). Sarcomatoid carcinoma of the stomach. A report of three cases with immunohistochemical and ultrastructural observations. Cancer.

[4] Xu F, Zou WB, Li XP (2013). Multiple carcinosarcomas of the esophagus and stomach. Oncol Lett.

[5] Ikeda Y, Kosugi S, Nishikura K (2007). Gastric carcinosarcoma presenting as a huge epigastric mass. Gastric Cancer.

[6] Kisluk J, Gryko M, Guzinska-Ustymowicz K, Kemona A, Kędra B (2013). Immunohistochemical diagnosis of gastrointestinal stromal tumors—an analysis of 80 cases from 2004 to 2010. Adv Clin Exp Med.

[7] Teramachi K, Kanomata N, Hasebe T, Ishii G, Sugito M, Ochiai A (2003). Carcinosarcoma (pure endocrine cell carcinoma with sarcoma components) of the stomach. Pathol Int.

[8] Izumi H, Yazawa N, Furukawa D (2016). Carcinosarcoma of the ampulla of Vater: a case report and literature review. Surg Case Rep.

[9] Yamazaki K (2003). A gastric carcinosarcoma with neuroendocrine cell differentiation and undifferentiated spindle-shaped sarcoma component possibly progressing from the conventional tubular adenocarcinoma; an immunohistochemical and ultrastructural study. Virchows Arch.

[10] Ro JY, Chen JL, Lee JS, Sahin AA, Ordóñez NG, Ayala AG (1992). Sarcomatoid carcinoma of the lung. Immunohistochemical and ultrastructural studies of 14 cases. Cancer.

[11] Randjelovic T, Filipovic B, Babic D, Cemerikic V, Filipovic B (2007). Carcinosarcoma of the stomach: a case report and review of the literature. World J Gastroenterol.

[12] Matsukuma S, Wada R, Hase K, Sakai Y, Ogata S, Kuwabara N (1997). Gastric stump carcinosarcoma with rhabdomyosarcomatous differentiation. Pathol Int.

